# A dataset of 200 structured product labels annotated for adverse drug reactions

**DOI:** 10.1038/sdata.2018.1

**Published:** 2018-01-30

**Authors:** Dina Demner-Fushman, Sonya E. Shooshan, Laritza Rodriguez, Alan R. Aronson, Francois Lang, Willie Rogers, Kirk Roberts, Joseph Tonning

**Affiliations:** 1 U.S. National Library of Medicine, NIH, 8600 Rockville Pike, Bethesda, MD 20894, USA; 2 UT Health School of Biomedical Informatics, 7000 Fannin St., Houston, TX 77030, USA; 3 Office of New Drugs, Center for Drug Evaluation and Research, U.S. Food and Drug Administration, 10001 New Hampshire Ave, Silver Spring, MD 20903, USA

**Keywords:** Data mining, Standards, Drug regulation, Data acquisition

## Abstract

Adverse drug reactions (ADRs), unintended and sometimes dangerous effects that a drug may have, are one of the leading causes of morbidity and mortality during medical care. To date, there is no structured machine-readable authoritative source of known ADRs. The United States Food and Drug Administration (FDA) partnered with the National Library of Medicine to create a pilot dataset containing standardised information about known adverse reactions for 200 FDA-approved drugs. The Structured Product Labels (SPLs), the documents FDA uses to exchange information about drugs and other products, were manually annotated for adverse reactions at the mention level to facilitate development and evaluation of text mining tools for extraction of ADRs from all SPLs. The ADRs were then normalised to the Unified Medical Language System (UMLS) and to the Medical Dictionary for Regulatory Activities (MedDRA). We present the curation process and the structure of the publicly available database SPL-ADR-200db containing 5,098 distinct ADRs. The database is available at https://bionlp.nlm.nih.gov/tac2017adversereactions/; the code for preparing and validating the data is available at https://github.com/lhncbc/fda-ars.

## Background & Summary

A standardised, validated searchable dataset containing information about labelled adverse reactions, i.e., adverse reactions present in Structured Product Labels (SPLs) for FDA approved drugs is needed to support such important activities as determining if an adverse reaction reported in clinical trials is caused by the study drug or a concomitant drug; conducting post-market surveillance for previously unobserved reactions; determining whether a drug could be repurposed (i.e., for a new indication); or finding patterns to predict drug interactions or other toxicity by pharmacologic class or similar chemical moieties.

Several attempts have been made to generate such a resource automatically from SPLs, one of the most comprehensive and authoritative sources of drug information. The widely used SIDER (Side Effect Resource) database combines natural language processing (NLP) and filtering techniques to automatically extract adverse drug reactions (ADRs) from SPLs^1^. SIDER is somewhat noisy: 1.2% of the extracted ADRs were indications^1^. NLP-based approaches might also miss out-of-vocabulary ADRs. The Adverse Drug Reaction Classification System (ADReCS) combines SIDER data with ADRs extracted directly from SPLs^2^. The ADRs in ADReCS are partially manually mapped to the Unified Medical Language System (UMLS) Metathesaurus (https://www.nlm.nih.gov/research/umls/) and to the Medical Dictionary for Regulatory Activities (MedDRA) (https://www.meddra.org/.) MedDRA is a standardised international medical terminology for regulatory communications in the registration, documentation and safety monitoring of medicinal products through all phases of the development cycle: from clinical trials to post-marketing surveillance. The UMLS integrates and distributes key biomedical terminologies and coding standards, including MedDRA. Mapping the ADRs to the UMLS facilitates interoperability, e.g., if an electronic health record contains ADRs encoded in another medical terminology, UMLS will provide a crosswalk from this terminology to MedDRA.

The mapping (normalisation) of adverse reactions in SPLs to a controlled vocabulary is extremely important in comparing ADRs across various sources of information. The European Medicines Agency (EMA), therefore, has developed the PROTECT ADR database that lists MedDRA terms for all ADRs of medicinal products authorised in the European Union (http://www.imi-protect.eu/adverseDrugReactions.shtml). EMA automatically mapped ADR terms to the MedDRA terminology and manually reviewed the results. Rules for extraction of ADRs from the SPLs were used in Structured Product Label Information Coder and Extractor that standardised ADRs to MedDRA^3^.

Although the above ADR databases have partially evaluated the accuracy of their extraction, to the best of our knowledge, no fully reviewed authoritative source of the size sufficient for development and evaluation of automated approaches to ADR extraction from SPLs exists. Our goal is, therefore, to develop such a rigorously validated resource, SPL-ADR-200db, to stimulate development of automated ADR extraction methods, facilitate community-wide evaluations, and provide a gold standard for future research purposes, for example, involving such sources as repoDB^4^.

SPL-ADR-200db provides both the fine-grained exhaustive manual annotations of the adverse reactions, and the mappings of the distinct reactions to the UMLS and MedDRA Preferred Terms (PTs) and Lower Level Terms (LLTs) validated by the National Library of Medicine and MedDRA editor representatives. SPL-ADR-200db consists of two parts: 1) a set of text documents fully annotated with ADRs, and 2) a database of distinct ADRs for each of the sections designated to report ADRs for each of the 200 drugs. The distinctions by section are important as the section indicates the clinical significance of the ADR (https://www.fda.gov/downloads/drugs/guidancecomplianceregulatoryinformation/guidances/ucm075057.pdf.) Some ADRs may be noted in more than one labelling section (https://www.fda.gov/downloads/drugs/guidancecomplianceregulatoryinformation/guidances/ucm075096.pdf.) A naïve automatic approach would be to label any untoward medical event in the designated sections as an ADR. In reality, the ADRs can be distinguished from indications, comorbidities, and background information provided in these sections only by understanding the immediate context in which they are mentioned, which explains why a human-curated resource is needed, as shown by Khare *et al.*^5^

**Figure 1:**
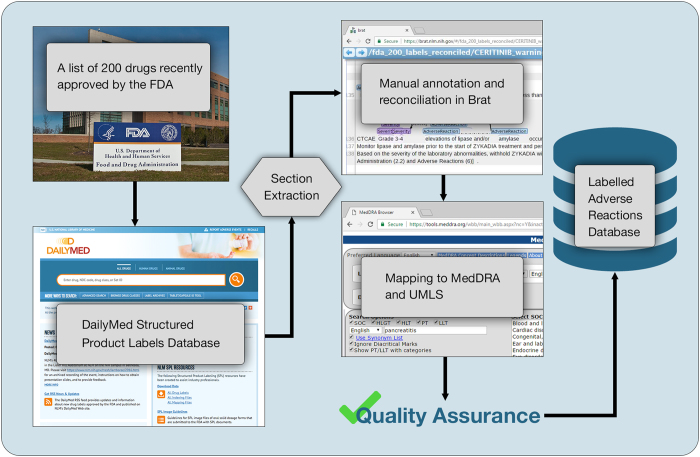
SPL-ADR-200db sources and curation process. The process consists of the following steps: 1) selecting the 200 most recently approved drugs 2) downloading the SPLs, 3) extracting the drug label sections designated to report ADRs, 4) annotating the extracted label sections, 5) extracting the distinct annotated ADRs and mapping these ADRs to the UMLS Metathesaurus and MedDRA, 6) quality assurance of the annotations and the mappings, and 7) generating the annotated dataset and the database of distinct labelled ADRs by SPL section.

SPL-ADR-200db can be used in any tasks involving extraction of adverse reactions from text and serves as an example for transforming SPL text into structured data for other purposes, such as extraction of drug-drug interactions, contraindications, or adverse reactions in specific populations. SPL-ADR-200db has enabled an open and transparent evaluation: the Text Analysis Conference (TAC) 2017 Adverse Drug Reaction Extraction from Drug Labels task (https://bionlp.nlm.nih.gov/tac2017adversereactions/.) A schematic overview of our approach to generation of the database is given in Fig. 1.

## Methods

The main steps for the construction of the database of labelled adverse reactions in the 200 drug labels were 1) selection of the 200 most recently approved drugs; 2) download of the Structured Product Labels (SPLs) in Extensible Markup Language (XML) format from the DailyMed site that maintains the labels: https://dailymed.nlm.nih.gov/dailymed/; 3) extraction of the drug label sections designated to report ADRs; 4) manual annotation of the text of the extracted sections using the *Brat* annotation tool^6^; 5) extraction of the distinct ADRs from the labels and manual mapping (normalisation) of these ADRs to the UMLS Metathesaurus and MedDRA; 6) quality assurance of the exhaustive annotations and the mappings; and 7) generation of the annotated dataset and the database of the distinct labelled ADRs by SPL section.

### Extraction of the drug label sections

In 2005, FDA started providing labelling information for prescription drugs on the Internet using SPLs in XML format. The SPL includes structured elements such as ingredients, as well as the human readable content organised into marked-up sections and subsections containing free text (https://www.fda.gov/downloads/ForIndustry/DataStandards/StructuredProductLabeling/UCM321876.pdf.) Major sections are defined by regulations and assigned Logical Observation Identifiers Names and Codes (LOINC) codes (https://loinc.org/.) As mentioned above, the distinctions by section are clinically important: the BOXED WARNING section of the label contains ADRs serious in proportion to the potential benefit from the drug (e.g., fatal, life-threatening or permanently disabling ADRs), whereas the WARNINGS AND PRECAUTIONS section contains ADRs that are serious or are otherwise clinically significant because they have implications for prescribing decisions or for patient management. The ADVERSE REACTIONS section includes ADRs of varying clinical significance, ranging from serious to minor and only information that would be useful to health care practitioners making treatment decisions and monitoring and advising patients.

Using the XML structure of the SPLs and the LOINC codes shown below, we have extracted the following sections, if present in a given SPL (the variations in the section naming are due to the changes to XML schema over the years):

- BOXED WARNING [34066-1]
- WARNINGS [34071-1]
- GENERAL PRECAUTIONS [34072-9]
- PRECAUTIONS [42232-9]
- WARNINGS AND PRECAUTIONS [43685-7]
- ADVERSE REACTIONS [34084-4]

### Exhaustive manual annotation of the section text

Manual annotation of the text required several decisions on: 1) the scope of annotation; 2) the tool to use for annotation; and 3) the annotation process.

For the scope and level of details for each adverse reaction, we had to decide whether to annotate specific drug doses that caused it; specific populations in which it occurred; the severity of the reaction; its comparison to the occurrences in the placebo arms of the trials; the prevalence, and many other factors. For this pilot annotation, we settled on annotating the ADRs, their severity and whether they were observed in animals or reported for the class of the drug or for the drug itself. More details are provided in the annotation guidelines: https://bionlp.nlm.nih.gov/tac2017adversereactions/AnnotationGuidelines_TAC2017ADR.pdf.

**Figure 2:**
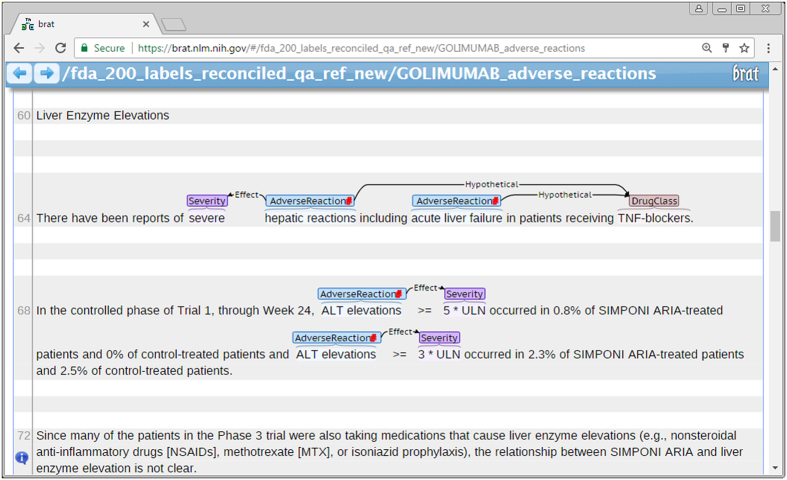
Exhaustive annotation of ADRs using *Brat.* The ADRs ‘hepatic reactions’ and ‘acute liver failure’ are attributed to the drug class of the label drug SIMPONI ARIA (golimumab), but not to the drug itself. The ALT elevations are annotated as asserted for the label drug and their severities are also annotated. The liver enzyme elevations are not annotated because they are mentioned as background information for the other drugs also taken by the patients in the Phase 3 of the trial. Note, that per FDA guidance, we annotated the abnormal results of the laboratory tests: ALT elevations. For some applications, this information might not be of interest. If desired, these ADRs can be excluded based on the mapping to the UMLS concept C1443982 [*ALT (SGPT) level raised*] that is of the semantic type Laboratory or Test Result.

We chose the *Brat* annotation tool that allows annotating text documents via a web browser. Although *Brat* allows assisted annotation, i.e., it will display automatically identified ADRs that can be manually edited, we chose to annotate the ADRs completely manually (i.e., from scratch) to avoid bias, e.g., missing the ADRs unmarked in the pre-annotation process^7,8^, as well as potential slow-down due to too many false-positives generated by a pre-annotation tool^9^. We annotated ADRs as events, which allows associating the details about the ADR to it as shown in Fig. 2. Brat stores annotations in stand-off format in the annotation files paired with the text files shown to the annotators. In the annotation files, the ADRs are presented as follows:

Event ID Event Name Start in the text End in the text Exact string

For example:

T35 AdverseReaction 3974 3991 hepatic reactions

To correct for the individual biases of the annotators, the annotation process was as follows: four annotators (two MDs, a medical librarian and a biomedical informatics researcher, all trained in biomedical annotation) annotated independently. Each label section of interest (BOXED WARNING, WARNINGS AND PRECAUTIONS, and ADVERSE REACTIONS) was doubly annotated. The disagreements between the annotators were reconciled in pairs. The remaining disagreements were resolved by all four annotators. Using F-score as measure of pairwise agreement between the annotators^10^, the agreement was good and improved during the process: the F-score was 74.9±0.9% on average for the first 100 SPLs and 85.5%±0.9% for the second half of the SPLs.

### Manual normalisation of the ADRs

We extracted 5,098 distinct ADRs from the Brat annotation files. We used the UMLS 2015AB REST API and the downloaded MedDRA 18.1 files to find perfect matches to the exact ADR strings. The primary terminology for FDA pre-marketing and post-marketing drug safety evaluation purposes is MedDRA. Therefore, we initially restricted the UMLS searches to MedDRA, and only used the appropriate semantic types from other UMLS sources if the ADR could not be mapped to MedDRA. The 1,383 perfectly matched MedDRA Preferred Terms (PTs) were split into four parts and reviewed manually by one of the annotators. The unmatched terms were also split into four parts, mapped manually by one of the annotators, and then the mappings were verified by another annotator. The disagreements were reconciled by all annotators.

In the process, we identified 114 ADRs that could not be mapped to MedDRA PTs, e.g., label text denoting ‘decreased embryo viability’. These terms were submitted to editor representatives from MedDRA for review. MedDRA editors suggested alternate mappings, some underspecified, for 39 unmapped terms and 2 annotations were deleted on their suggestions. 73 terms remain unmapped.

We also identified 102 additional underspecified terms, e.g., we could only map the label text ‘flexor tendon ruptures’ to the MedDRA PT 10043248 ‘Tendon rupture’. We submitted these additional 102 terms to the MedDRA editors. After their review, 83 ADRs remained underspecified, while 19 had alternate mappings suggested by the editors. For example, we coded the ADR ‘renal tubular injury’ to the MedDRA PT 10061481 ‘Renal injury’, whereas MedDRA editors suggested using ‘Renal tubular dysfunction’ instead, and further proposed to add ‘Renal tubular injury’ as a Preferred Term to the next version of MedDRA dictionary.

### Quality assurance (QA)

During reconciliation, we noticed that for a list of ADRs, common modifiers are often mentioned only once with the first ADR. For example, ‘*injection-site reactions (e.g., injection-site rash, erythema)*’. Initially, we sometimes annotated such listed ADRs on their own, but in the QA process we extended annotations to the fully stated ADRs, e.g., ‘*injection-site erythema*’.

To ensure consistency in mapping similar terms, e.g., plural forms, abbreviations, different word order, or different levels of abnormal laboratory results, we analysed the lists of terms ordered alphabetically, by MedDRA identifiers and by UMLS identifiers. This analysis allowed us to detect two types of discrepancies: mapping an ADR to the same MedDRA PT, but to a different UMLS term, and mapping the same ADR to different MedDRA PTs. None of the mappings were wrong. Most of the disagreements in having the same MedDRA PT but different UMLS terms occurred because one group of annotators mapped the lower level MedDRA terms (LLT) that better matched the ADRs in text, and the other group mapped the PT instead. For example, ‘changes in creatinine level(s)’ was mapped to the MedDRA LLT 10011359 ‘Creatinine abnormal,’ for which the PT is 10005481 ‘Blood creatinine abnormal’, and subsequently to UMLS C0740471 ‘Creatinine abnormal NOS’ and C0853746 ‘Blood creatinine abnormal’, respectively. We added a rule to the guidelines to use the LLT mappings to the UMLS in such cases. Note that ‘changes in creatinine level(s)’ were annotated as abnormal only if indicated in context. For example, in ‘Patients treated with TREANDA may also have changes in their creatinine levels,’ ‘changes in creatinine levels’ were annotated as the following sentence in the same section states: ‘Clinically important chemistry laboratory values that were new or worsened from baseline and occurred in >1% of patients at Grade 3 or 4, in NHL patients treated in both single arm studies combined were hyperglycemia (3%), elevated creatinine (2%), hyponatremia (2%), and hypocalcemia (2%).’

For mapping to different MedDRA PTs, the differences were mostly in the nuances. For example, MedDRA terminology for specific administration site reactions is very rich and provides terms for application, instillation and injection site reactions, e.g., Preferred Terms such as ‘Application site blister’, ‘Instillation site blister’, and ‘Injection site blisters’. In some cases, administration type was not clear from the content of the ADR section alone, and we had to consult administration instructions while reconciling such discrepancies.

Finally, in many cases the ADRs are somewhat vague and use such terms as ‘event’, ‘reaction’ or ‘risk’, e.g., ‘cerebrovascular events’, ‘cerebrovascular reactions’, ‘cerebrovascular risk’. Prior to the mapping process, we were not aware that these terms could not be coded to MedDRA exactly. As a result, we sometimes mapped such expressions to disorders, sometimes to symptoms, and sometimes to accidents. In the quality assurance process, we established a hierarchy of increasing severity to follow depending on the availability of the terms in MedDRA: exact match → risk → symptom →disorder.

### Generation of the final databases

The final SPL-ADR-200db database was generated in two formats: 1) a dataset of 200 pairs of text files and *Brat* annotation files, and 2) a database of distinct asserted ADRs for each ADR section of each of the 200 drugs in comma-separated value (CSV) format. Although both forms of SPL-ADR-200db present the same coding information, the paired text files format is intended for development of NLP applications, whereas the CSV format (shown in Table 1) is intended to serve as the first publicly available set of labelled ADRs, and as an example for similar datasets in the future.

**Figure 3:**
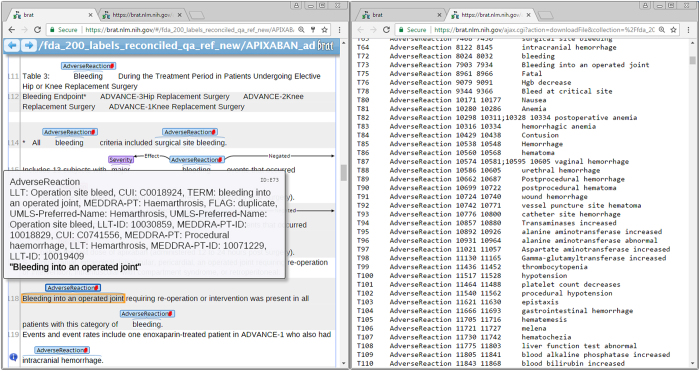
SPL-ADR-200db in Brat format. The left pane shows the human readable text files and the annotations in Brat. The right pane shows the corresponding annotation file that contains information about each ADR event: the offset of the mention in the text files, the relations, and the mappings to MedDRA and UMLS terms.

To facilitate development of NLP applications, and as a final quality assurance step, we augmented *Brat* annotation files with MedDRA and UMLS codes as shown in Fig. 3.

### Code availability

Code for extraction of the ADR sections from Structured Product labels is available at https://github.com/lhncbc/fda-ars.

## Data Records

We downloaded the DailyMed SPL database from https://dailymed.nlm.nih.gov/dailymed/spl-resources-all-drug-labels.cfm on September 29, 2015. The UMLS files containing medical terminology used in the normalisation steps can be found in https://download.nlm.nih.gov/umls/kss/2017AA/umls-2017AA-active.zip. We uploaded to Open Science Framework SPL-ADR-200db training set files (Data Citation 1), the test files (Data Citation 2) in Brat annotation format, and the comma-separated value files containing adverse reactions by sections (Data Citation 3).

## Technical Validation

We cross-validated the Brat annotation files and the comma-separated value (CSV) files that contain distinct ADRs for each SPL section by assuring that all mention-level annotations are accounted for in the CSV files, and that all ADRs in CSV files correspond to at least one mention-level annotation.

The *Brat* annotation files were validated in generating the training and test sets for the TAC 2017 ADR challenge in XML format. The XML files combine the text files extracted from SPLs with the mention level annotations from *Brat* annotation files and MedDRA and UMLS terms and codes for each mention.

Usability of the SPL-ADR-200db files for evaluating ADR extraction system was validated using MetaMap^11^ to extract ADRs, and evaluating the results under several conditions known to maximise recall or precision. We obtained the expected variation in these metrics: Precision of 69.90% with 59.25% recall, when precision was maximised, and 62.36% precision with maximum recall of 71.65%.

## Usage Notes

To ensure that all researchers understand how to use the data, we provide instructions at https://bionlp.nlm.nih.gov/tac2017adversereactions/.

## Additional information

**How to cite this article:** Demner-Fushman, D. *et al.* A dataset of 200 structured product labels annotated for adverse drug reactions. *Sci. Data* 5:180181 doi:10.1038/sdata.2018.1 (2018).

**Publisher’s note:** Springer Nature remains neutral with regard to jurisdictional claims in published maps and institutional affiliations.

## Supplementary Material



## Figures and Tables

**Table 1:** SPL-ADR-200db format for presenting distinct ADRs by SPL section.

**DB column**	**Example1**	**Example2**	
Drug ID	4e338e89-3cf2-48eb-b6e2-a06c608c6513	e9481622-7cc6-418a-acb6-c5450daae9b0	
Drug name	Zytiga	Eliquis	
Section code	34084-4	34084-4	
Section name	ADVERSE REACTIONS	ADVERSE REACTIONS	
Sample ADR in text	high total bilirubin	bleeding into an operated joint	
MedDRA PT	Blood bilirubin increased	Haemarthrosis	Procedural haemorrhage
MedDRA PT ID	10005364	10018829	10071229
MedDRA LLT	Bilirubin total high	Hemarthrosis	Operation site bleed
MedDRA LLT ID	10004697	10019409	10030859
UMLS Preferred Name	Elevated total bilirubin	Hemarthrosis	Operation site bleed
UMLS CUI	C0741494	C0018924	C0741556
Flag		Duplicate	Duplicate
Example 2 shows a record with a duplicate flag that indicates two terms are needed to describe the ADR ‘bleeding into an operated joint.’			
